# MHA Herbarium: Eastern European collections of vascular plants

**DOI:** 10.3897/BDJ.8.e57512

**Published:** 2020-10-23

**Authors:** Alexey P. Seregin, Nina Yu. Stepanova

**Affiliations:** 1 Lomonosov Moscow State University, Moscow, Russia Lomonosov Moscow State University Moscow Russia; 2 M.V. Lomonosov Moscow State University, Moscow, Russia M.V. Lomonosov Moscow State University Moscow Russia; 3 Tsitsin Main Botanical Garden, RAS, Moscow, Russia Tsitsin Main Botanical Garden, RAS Moscow Russia

**Keywords:** occurrence, specimen, herbarium, database, digitisation, georeferencing, collections

## Abstract

**Background:**

World herbaria with 387.5M specimens (Thiers 2019) are being rapidly digitised. At least 79.9M plant specimens (20.6%) are already databased throughout the globe in the standard form of GBIF-mediated data. The contribution of smaller herbaria has been steadily growing over the last few years due to cost reduction, usage of platforms and solutions developed by the leaders. A web-resource the Moscow Digital Herbarium (Seregin 2020b) was launched by the Lomonosov Moscow State University in October, 2016 for publication of specimens imaged and databased in the Moscow University Herbarium (MW). As of 31 December 2018, the web-portal included 968,031 images of 971,732 specimens digitised in MW. This dataset is available in GBIF (Seregin 2020). The global trend is largely the same in Russia, where a dozen herbaria started to scan their holdings after imaging of the nation’s second largest herbarium (Kislov et al. 2017, Kovtonyuk et al. 2019, Seregin 2020a). In 2019, we started to use Moscow Digital Herbarium as a web-repository for digitised herbarium specimens from some Russian collections, starting with the Herbarium of Tsitsin Main Botanical Gaden, Russian Academy of Sciences (MHA). Due to this, a single-university system became a multi-institutional consortium in April 2019 (Seregin 2020a). The dataset of the Moscow collections and partly of the Eastern European collections of the MHA Herbarium is now available in GBIF (Seregin and Stepanova 2020).

**New information:**

MHA Herbarium imaged 64,008 specimens from Moscow Region and partly from other regions of Eastern Europe at 600 dpi and provided key metadata. These data are now fully available in the Moscow Digital Herbarium and GBIF. Complete georeferencing of the specimens from the City of Moscow was a key task in 2020. As of May 2020, 50,324 specimens, including 49,732 specimens from Russia, have been georeferenced (78.6%) and 39,448 specimens have fully-captured label transcriptions (61.6%). Based on these data, we give a detailed overview of the collections including spatial, temporal and taxonomic description of the dataset.

## Introduction

The official name of the collection is the Skvortsov Herbarium of the Main Botanical Garden, Russian Academy of Sciences (acronym MHA). In 2020, the Herbarium was named after the well-known Russian botanist Alexey Konstantinovich Skvortsov (1920–2008), who was the scientific supervisor of the MHA Herbarium for 36 years.

The Herbarium was launched soon after the founding of the Main Botanical Garden of the Academy of Sciences of the USSR in 1945. Initially, some minor collections of dry plants were stored in workrooms of the staff. In 1958, the Herbarium received a hall of 280 m^2^ in the newly-constructed main lab building. A group headed by V.N. Voroshilov formed the herbarium staff. Upon formal establishment, the MHA Herbarium received an almost complete set of exsiccates “Herbarium of the Flora of the USSR” from the Komarov Botanical Institute (Leningrad) and all botanical collections from the Timiryazev Institute of Plant Physiology (Moscow), including duplicates of important Moscow collections by D.P. Syreyshchikov, the first curator of the Moscow University Herbarium. These initial holdings were supplemented by the collections from Voronezh and Moscow Oblasts by V.N. Voroshilov, B.M. Kulkov and V.A. Shtamm (Stepanova et al. 2020).

In 1966, A.K. Skvortsov became the scientific supervisor of the MHA Herbarium. The main vectors of the Herbarium development were formed in this time: “Our collections should provide:

1. orientation in the flora as a source of the material for introduction;
2. documentation of the introduction activities.

The location of the herbarium in the center of European Russia obliges us to create a regional herbarium” (Skvortsov and Proskuryakova 1973). Skvortsov formed the main sections of the Herbarium—the Russian Far East, Siberia, Middle Asia, the Caucasus, the Moscow Region, the European part (European Russia and adjacent republics of the former USSR), the Crimea; General Herbarium (foreign countries); Herbarium of Introduction; Dendrological Herbarium; type collection; Skvortsov’s personal herbarium (taxonomic collections of *Salix*, *Populus*, *Betula*, *Epilobium*, as well as materials on the flora of Middle Russia and Lower Volga).

Some Russian-language references describe the main milestones in the history of the MHA Herbarium (Skvortsov and Proskuryakova 1973, Skvortsov 1977, Belyanina and Makarov 1994, Skvortsov and Belyanina 2005, Ignatov et al. 2010, Ignatov 2015, Stepanova et al. 2020). As of January 2020, the MHA Herbarium holds 615,223 specimens of vascular plants and ca. 70,000 specimens of bryophytes. The general structure of the MHA Herbarium is given in Table 1. The annual growth of the collections since 2015 was ca. 5,900 accessions of vascular plants and ca. 2,000 accessions of bryophytes. This is the fourth largest herbarium of Russia after the Komarov Institute, RAS in St. Petersburg (LE), Moscow State University (MW) and the Joint Novosibirsk Herbarium, RAS (NS + NSK).

The Herbarium of vascular plants is located in two halls (334 m^2^) in the main lab building of the Garden. Duplicates and unmounted backlog are stored in several rooms (120 m^2^) at Botanicheskaya Street, 33-4 within a ten minute walk from the main building. The Herbarium of bryophytes is also stored at Botanicheskaya Street, 33-4 in several rooms (180 m^2^).

Currently, the MHA Herbarium has 12 staff members (of which six are working with vascular plants). There are eight curators and researchers, a mounter and three employees who are digitising and filing the specimens. The staff members conduct field research across Russia in Tver, Tula, Kaluga, Belgorod, Rostov, Saratov, Volgograd, Orenburg Oblasts, Kalmykia, Dagestan, Ingushetia, Stavropol Krai, Yakutia etc. The total duration of expeditions is ca. 240 person-days per year. Fresh collections by the employees form 70% of new accessions. Other accessions come from exchange, gifts and old backlog.

Recent digitisation activities in Moscow and Eastern European sections allow us to detail the list of collectors, the time and place of their work, the number of the collected specimens and their taxonomic composition.

**1. Moscow section** holds 49,621 specimens and covers two subjects of the Russian Federation—the City of Moscow and Moscow Oblast. The section is completely imaged and curated as a separate unit due to the geographical location of the Herbarium and high intensity of field research in the area.

**2. Eastern European section** covers plant collections from European Russia, the Urals, the Baltic countries, Belarus, Ukraine, Moldova and Western Kazakhstan (west of the Ural River). The section does not include Moscow Region, the Caucasus and the Crimea. Today, the section contains 101,034 specimens, incl. 14,288 imaged specimens (14.1%). Thus, statistics on taxonomic and temporal coverage, collectors and collection dates for this section are based on a 14-percent sample that covers pteridophytes, conifers and most monocots.

## Project description

### Title

"Flora of Moscow" Information System on Moscow Digital Herbarium web-platform (research project #19-34-70018).

The MHA Herbarium holds vast collections from the Moscow metropolitan area (City of Moscow and Moscow Oblast) collected in the last 70 years, whereas the Moscow University Herbarium (MW) holdings are fairly evenly distributed in time over a period of 200 years. Altogether, MW and MHA have 130,000+ specimens from the Moscow Region, which make it the most densely-sampled territory across Russia. The idea of the research proposal was to digitise and precisely georeference this large dataset for proper understanding of changes in the flora around the City of Moscow through time and space. From March to October 2019, the MHA Herbarium team imaged 49.7K herbarium specimens of vascular plants at 600 dpi using Microtek 1600 Object Scan. In the next few months, 78.6% of them were georeferenced.

Further, the MHA Herbarium published online 15K images of its Eastern European collections (imaged earlier in 2017-2018), which are especially strong in the semi-arid flora of the Lower Volga Region. The MHA Herbarium collections are fully available in GBIF, Moscow Digital Herbarium and newly-established "Flora of Moscow" website (https://moscow.depo.msu.ru). At the moment, MHA Herbarium is the second largest imaged herbarium of Russia.

## Sampling methods

### Step description

To schedule and perform the digitisation of the MHA Herbarium, we used five key stages by Nelson et al. (2012):

- pre-digitisation curation and staging,
- specimen image capture,
- specimen image processing,
- electronic data capture,
- georeferencing specimen data.

**1. Pre-digitisation curation and staging**

The section curator reviews all incoming physical accessions for meeting the basic requirements of the herbarium specimen. A specimen should be a high-quality dried plant (or several individuals) with a label bearing identification, collection site, habitat, collection date and collector. After that, unmounted new material is frozen at a temperature of –30°C for 14 days as a quarantine procedure against specific herbarium pests and then mounted. New collections are counted (and listed in the collection journal) right after mounting. Sorting and incorporation of new material takes place once a year, usually in the autumn-winter period. Right before imaging, pre-ordered self-adhesive barcodes with an acronym and a seven-digit number (e.g. MHA 0 002 094) were attached to the herbarium sheet.

**Eastern European section.** In December 2017, with the purchase of a specialised scanner Microtek ObjectScan 1600, we began the imaging of vascular plants in the MHA Herbarium. Since the specimens from European Russia and adjacent states constitute the largest and most used section of the Herbarium, we decided to start imaging from this section. If there were two or more taxa on a single sheet, they were remounted on separate sheets.

**Moscow section.** From March to October 2019, the Moscow section of the Herbarium was imaged in line with the work under the RFBR grant "Information system Flora of Moscow on the platform of the Moscow Digital Herbarium" (under A.P. Seregin). The imaging of the Eastern European section was suspended for this time. Within the framework of the project, N.M. Reshetnikova (MHA Herbarium) and S.R. Mayorov (Moscow University) thoroughly revised the taxonomy of all Moscow specimens. After that, we checked the nomenclature on the folders and corrected it against the accepted backbone.

**2. Specimen image capture**

Specimens were imaged in accordance with international standards with a resolution of 600 dpi and a colour checker (24 colours). After scanning, each image was automatically renamed according to the barcode served as an unique identifier. In total, 14,274 specimens of the Eastern European section were digitised in 2017–2018. Imaged Eastern European collections at that time were stored on external discs without online access.

The Moscow section was scanned more intensively under the time limit from March to October 2019. Every day, two to three operators worked on the single scanner in shifts. For each shift lasting four to five hours, 140–160 specimens were digitised. Thus, 300–400 specimens were imaged per day per scanner. In total, the herbarium team imaged 49,621 specimens within eight months and completed the mission.

During the imaging, we encountered a number of minor issues:

- Some specimens have large plants covering partly or fully the label text. The specimens were imaged as they are, whereas the labels will be captured not from the image, but from the physical specimen later.
- Sometimes two different species were mounted on a single sheet. In such cases, if possible, the specimens were remounted on to two sheets. If the remounting was impossible or impractical, the single sheet was scanned, but the image was duplicated and each file was assigned an additional digit ("-1" or "-2") to facilitate unique identifiers for each species.
- Labels of a larger size widely used in the exsiccates "Herbarium of the flora of the USSR" were often folded during mounting. We tried to remount such labels to make text fully available on images, but in some cases, the label partly covered the plant.
- In some cases, two or more parts of the same large plant were mounted on several sheets bearing a single label and further notes like “sheet #2”, “sheet #3” etc. These sheets were initially inserted into the cupboards after being fastened with a removable paper clip. However, they have been mixed over time with other specimens, so now it is impossible to trace the correct label for these multiple “sheets #2”.

**3. Specimen image processing**

While scanning, the operator started a new directory for every species and named it against a folder name. Before uploading the images into the Moscow Digital Herbarium, the structure of the directories was converted into a table of metadata. Thus, for each accession, the initial metadata included ID (barcode identifier), taxon name from folder without taxonomic authors and the geographic code of the area.

The taxon name, according to the protocols of the Moscow Digital Herbarium, was automatically matched with the latest version of the Catalogue of Life (CoL), from which the complete accepted name, synonymy and hierarchical list of supraspecific taxa were downloaded for every entry.

Publication of images with brief metadata is a powerful tool for rapid online access to the scanned herbarium collections. This approach was largely used in Paris where the largest herbarium of the world was imaged and published online (Le Bras et al. 2017). Similar protocols were adopted in Edinburgh and Moscow University (Haston et al. 2012, Seregin 2016, Seregin 2018).

After online publication of the Moscow Region specimens in the Moscow Digital Herbarium and GBIF, other sections of the MHA Herbarium will undergo the same procedure. Thus, to date, 64008 images of specimens of vascular plants from the MHA Herbarium are available online.

**4. Electronic data capture**

After online publication of the images and associated brief metadata, we link the records with existing full-label data capture of 7,087 specimens of the Moscow section (14.3%) made earlier by T.G. Nosova and I.A. Kravtsov in the form of a Microsoft Excel spreadsheet. For the remaining 42,534 specimens, the operators of the Moscow Digital Herbarium entered mandatory metadata—the collection date, the first collector, curatorial area and coordinates (if present on the label).

Similarly, a table with full-label data capture for 11,716 specimens from Eastern Europe (82% of the scanned ones) made by E.A. Karakina and B.L. Oshovskaya was uploaded as well. For the remaining 2,572 specimens, the operators of the Moscow Digital Herbarium and employees of the Garden entered additional mandatory metadata.

Thus, the minimum obligatory set of metadata available for all digitised specimens of the MHA Herbarium in this dataset include barcode ID, complete taxonomic information, collection date, the first collector, curatorial area and geographical coordinates (if available on the label). Additionally, 18,803 specimens had full-text inscriptions of labels (29.4%) due to earlier efforts.

Further full-text data capture was carried out by the operators of the Moscow Digital Herbarium for specimens collected within the City of Moscow (15,982 specimens). An operator entered the label data from the scanned image into an Microsoft Excel spreadsheet with 30 standard fields (including some pre-filled ones to avoid mistakes). Additionally, a commercial partner under the GBIF contract (2019) made full-text transcriptions of 4,617 specimens from the City of Moscow and Moscow Oblast.

After data entry, the scientific supervisor of the Moscow Digital Herbarium checked the spreadsheets for technical issues by a set of automatic, semi-automatic and manual operations. The IT-team, using the data migrator programme, then converted data from the Excel spreadsheet to the PostgreSQL database of the Moscow Digital Herbarium for further data storing and retrieving. This stage also includes some automatic checks of data consistency.

As of May 2020, the full text of labels has been entered for 39,448 specimens of the MHA Herbarium (61.6% of the imaged ones)—27,783 specimens of the Moscow section and 11,665 specimens of the Eastern European section. Full-text label transcriptions help to optimise the further georeferencing by combining labels with identical text into groups.

**5. Georeferencing specimen data**

The operators of the Moscow Digital Herbarium and the Garden employees carried out manual georeferencing with further implementation of the ISTRA system (Intellectual System of Toponymic Reading and Attribution), several lines of the code being written in JAVA. This code is integrated into the Moscow Digital Herbarium and unavailable as a stand-alone product.

The first algorithm of the ISTRA system combines the specimens into the groups according to the matching of the captured label text. In this case, there are two options for combining—complete matching mode and letters-only mode. The results do not differ in accuracy from the manual georeferencing. The second algorithm of the ISTRA system forms the specimen groups according to the matching of three fields—collection date, collector’s surname and curatorial area. Within the walking-day route, the standard georeferencing accuracy in most cases does not exceed 5 km. Further data refinement will help us to replace automatic georeferencing with the more accurate manual one.

In both cases, the operator inserts the coordinates manually and the system sets the coordinates automatically for all specimens of the group. The first algorithm takes precedence over the second one. In all cases, we save the log file and note the georeferencing method in the form of the standard disclaimers:

- captured from the label;
- set manually by the operator;
- set automatically by matching of the label text;
- set automatically by matching of the collection date and collector.

Manual georeferencing is carried out using standard e-cartographic libraries (Yandex.Maps, Google Maps, Wikimapia, SAS.Planet etc.) for modern specimens, whereas historic collections are georeferenced using the libraries of scanned maps (etomesto.ru, loadmap.net etc.) following the principle “collection date = map date”. Coordinate precision (rounded to 100 m) is set and stored for each manual georeferenced point.

Complete georeferencing of the specimens from the City of Moscow was a key task in 2020 for the Moscow University team (according to the Moscow project), whereas employees of the MHA Herbarium georeferenced specimens from Moscow Oblast and Eastern Europe (starting with the most prolific collectors). In total, 50,324 specimen have been georeferenced (74%), including 49,732 specimens from Russia.

For 7,414 specimens, the coordinates were taken from the label (14.7% of the number of georeferenced ones), for 10,849 specimens (21.6%), they were set manually and for 32,061 specimens (63.7%), they were calculated automatically using the ISTRA system.

## Geographic coverage

### Description

The Eastern European section of the MHA Herbarium has its focus on European Russia (Table 2). The most sampled areas are the Moscow Region, Lower Volga, Central and Central Forest-steppe Regions (Table 3), the areas intensively studied by the Garden staff. Initially, these Regions were inextricably linked with the activities of A.K. Skvortsov, whose fruitful initial collections often formed a solid basis for the later extensive floristic research.

Moscow Region forms its own section in the MHA Herbarium. This is due to the location of the Garden in the City of Moscow. One of the initial missions of the Herbarium was precise documentation of the local flora, including long-term observations of both native and alien plants. Based on these materials, standard flora and checklists were published by the Garden staff in collaboration with Moscow University (Voroshilov et al. 1966, Ignatov et al. 1990, Mayorov et al. 2012, Mayorov et al. 2020).

The Lower Volga Region was one of the focus areas for A.K. Skvortsov, his graduate students and the Herbarium employees. This activity resulted in the published volumes of the “Flora of the Lower Volga” (Skvortsov 2006, Reshetnikova 2018). The Central Region is also well-represented in the collection due to another long-term floristic interest of A.K. Skvortsov, which was later continued by the Herbarium staff who published “Kaluga Flora” (Reshentnikova et al. 2010). Belgorod Oblast in the Central Forest-steppe Region is a new area of the research headed by N.M. Reshetnikova. Other Regions are less represented and are not as complete and thorough. Usually, these are either collections from various field trips of the Garden staff or gifts.

Geographical coordinates for the dataset frame are given below.

### Coordinates

44.5 and 77 Latitude; 19.5 and 69.5 Longitude.

## Taxonomic coverage

### Description

The dataset covers vascular plants of Eastern Europe, both native and alien species. There are also some specimens of cultivated plants, especially from the Moscow Region. The Moscow section is completely digitised and can provide figures on the taxonomic representation of the MHA Herbarium collections, whereas 14% of the the Eastern European section has been digitised and, therefore, information on its taxonomic composition only shows which families have been digitised so far.

Until 2017, the taxonomic backbone of the MHA Herbarium was the standard checklist by Czerepanov (1995). In some cases, the section curators could deviate from this source depending on their taxonomic expertise. V.D. Bochkin in the Moscow section and N.M. Reshetnikova in the Eastern European section adjusted the deviations. In 2019, with the preparation of the Moscow collections for imaging, we revised the names on folders against The Plant List (TPL), superseded shortly by Plants of the World Online (POWO). A similar approach was subsequently used before imaging of the Eastern European holdings with some deviations emerging from either the standard regional flora or new taxonomic monographs (Pimenov and Ostroumova 2012, Tzvelev and Probatova 2019, etc.).

**Moscow section.** It includes 2,261 species and hybrids from 773 genera and 138 families. The flora of the Moscow Region (the City of Moscow and Moscow Oblast) currently enumerates 2,363 taxa of vascular plants, including both wild and unintentionally-introduced plants (Shcherbakov and Lyubeznova 2018). We could explain a high diversity of the Moscow collections by the incorporation of purely-cultivated plants practised by the researchers of the alien flora (in case these species were found outside of cultivation). The section also includes some reference specimens of the Main Botanical Garden exposition. For taxonomic coverage across the leading taxa, see Table 4, Table 5 and Table 6

**Eastern European section.** In this section, 14% of the collections have been digitised so far, therefore Table 7, Table 8 and Table 9 show not the taxonomic diversity of the section, but an overview of imaged specimens. The specimens were scanned one by one following the order of the physical collection—pteridophytes, gymnosperms and angiosperms following Engler’s system against the standard catalogue (de Dalla Torre and Harms 1900). Thus, Pteridophyta, Equisetophyta, Lycopodiophyta, Pinophyta and monocots from *Typha* to *Scirpus radicans* (Dalla Torre's numbers from 1 to 468) were imaged. In addition, the genus *Crataegus* (Rosaceae) was digitised out of sequence.

### Taxa included

**Table:** 

Rank	Scientific Name	
phylum	Tracheophyta	

## Traits coverage

### Data coverage of traits

PLEASE FILL IN TRAIT INFORMATION HERE

## Temporal coverage

### Notes

The Moscow and East European sections of the MHA Herbarium were launched in the second half of the 20th century and collections continue to grow in the 21st century. The mean year of collection for the digitised specimens of the entire MHA Herbarium is 1976. The mean collection date shows in which regions the Herbarium was currently active and from which areas, in particular, there is fresh material for DNA studies or adequate collections of recently-spreading alien species. From the majority of regions, new collections have come evenly since the foundation of the MHA Herbarium. They have an average collection date of 1975–1978 (Table 10).

In recent decades, collections came mainly from four Regions—Rostov Oblast, Lower Volga Region, Central Region (mainly Kaluga Oblast) and Central Forest-Steppe Region (mainly Belgorod Oblast). These are the places of the fieldwork of the current Herbarium employees, as well as the above-mentioned expeditions across the Lower Volga Region of the 1990s. On the contrary, there have been no significant accessions from Ukraine, Belarus, Moldova, Estonia, Latvia, as well as some regions of European Russia in recent decades.

**Figure 1.**
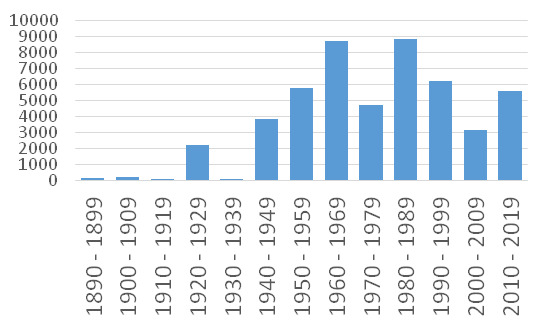
Temporal distribution of specimens in Moscow section, MHA Herbarium.

**1. Moscow section.** In this section, 49,550 specimens have collection date after 1890. Their temporal distribution over decades is given in Fig. 1.

The peak of 1920s resulted from the transfer of earlier collections by D.P. Syreishchikov. The original collections of the Moscow section of the MHA Herbarium dated back to the period 1945–2018 with two peaks of major accessions—1960s and 1980s.

In the 1960s, the main collections came from V.A. Shtamm (1,709 specimens), A.K. Skvortsov (1,370), V.V. Makarov (1,314), G.P. Rysina (831), E.I. Kurchenko (549), A.P. Khokhryakov (445), A.A. Nekrasov (356), E.E. Gogina (181), V.S. Drozdova (153) and N.K. Shvedchikova (143). This was the time of active studies of the native flora and the publication of the standard guide by Voroshilov et al. (1966), still the only monograph on the native flora of the Moscow Region.

In the 1980s, the most important Moscow collections were gathered by the MHA Herbarium employees M.S. Ignatov (2,716 specimens), V.D. Bochkin (2,585), V.V. Makarov (1,375), A.N. Shvetsov (577) and A.K. Skvortsov (512). Lesser contributions were made by V.B. Kuvaev (391), L.A. Deistfeldt (117), N.V. Kostyleva (108), A.E. Matsenko (91) and A.V. Shcherbakov (65). In this period, intensive studies of the alien flora of the Moscow Region resulted in the checklist by Ignatov et al. (1990). This paper became the foundation for the further study of invasive plants around Moscow (Mayorov et al. 2012, Mayorov et al. 2020).

**2. Eastern European section**

**Figure 2.**
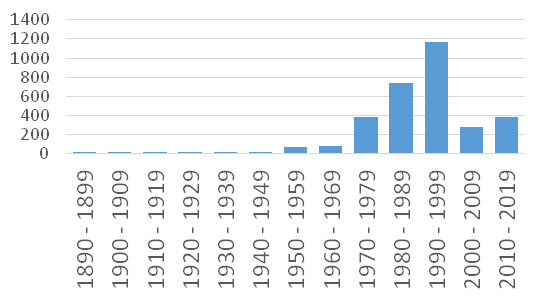
Temporal distribution of digitised specimens from Lower Volga Region, MHA Herbarium.

**2.1. Lower Volga Region.** At the moment, 3,244 specimens from this Region have been digitised so far. We assume that the total volume of collections from the Lower Volga is 23,170+ specimens. Figures given below are based on 3,145 digitised specimens (14% of the collection volume) having a collection date after 1890. Their temporal distribution over decades is given in Fig. 2.

Notable collections from the Lower Volga began to arrive in the mid-1970s, but the peak of the major accessions stretched over the 1980s and 1990s. Especially large collections were made in 1982, 1986, 1989–1990 and 1993–1994.

In the 1980s, the major collections came from N.B. Belyanina (2500 estimated number of specimens/350 digitized), V.D. Bochkin (990/139), V.A. Sagalaev (670/94) and G.Yu. Klinkova (460/64). In the 1990s, the most important collections from the Lower Volga were accessed from V.A. Sagalaev (2,080/291), G.Yu. Klinkova (1,940/271), V.D. Bochkin (1,470/206), I.A. Shantser (830/116), A.K. Skvortsov (790/110) and S.R. Mayorov (230/41). These figures are based on the senior collectors mentioned in the labels, but in 1993, a top-record year, an expedition supported by the U.S. National Geographic Society collected at least 5,100 specimens (716 digitised) during a many-month trip across the Lower Volga Region and Western Kazakhstan performed by V.A. Sagalaev, G.Yu. Klinkova, I.A. Shantser, V.D. Bochkin, A.K. Skvortsov, M.Yu. Polonskaya, M.V. Kostina, V.V. Dzhanaeva and others.

**Figure 3.**
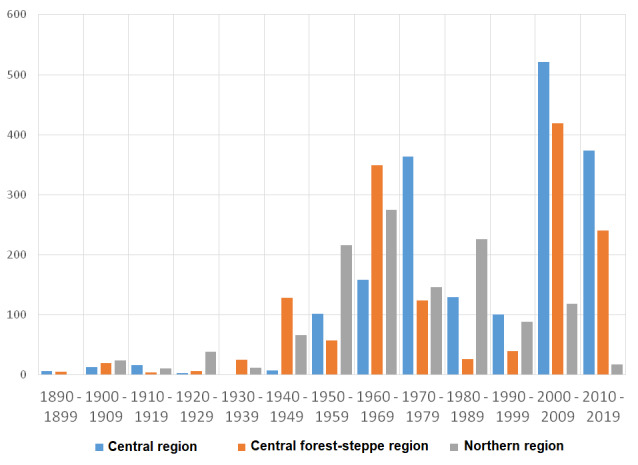
Temporal distribution of digitised specimens from the Central, Central Forest-steppe and Northern Regions, MHA Herbarium.

**2.2. Other Eastern European regions.** Amongst other Eastern European collections, the most noticeable are those from the Central Region (especially Kaluga Oblast) (12,940 estimated number of specimens/1,812 digitised), the Central Forest-steppe Region (especially Belgorod Oblast) (10,420/1,459) and the Northern Region (8,990/1,259). Their temporal distribution over decades is given in Fig. 3.

Accessions from the **Central Region** have two peaks—in the 1970s (especially 1971, 1974) and in 2000–2010s (especially 2003, 2007, 2014). In the 1970s, the main collections were received from V.V. Makarov (820 estimated number of specimens/115 digitised) and A.K. Skvortsov (290/41) and in the 2000–2010s from N.M. Reshetnikova (2,600/364), A.P. Seregin (940/132), A.V. Krylov (740/104) and A.A. Shmytov (320/45). The most sampled area is Kaluga Oblast which has been intensively studied by the Herbarium staff. This resulted in the publication of the standard regional flora (Reshentnikova et al. 2010).

The collections from the **Central Forest-steppe Region** have two peaks—in the 1960s (especially 1966, 1968) and in the 2000s (especially 2006-2008). In the 1960s, the main collections were acquired from V.V. Makarov (1,510 estimated number of specimens/211 digitised), A.P. Khokhryakov (330/46) and A.K. Skvortsov (230/32) and in the 2000s from N.M. Reshetnikova (1,410/197), A.K. Mamontov (790/110) and A.P. Seregin (340/47).

Accessions from the **Northern Region** are distributed more evenly across decades. Two peaks can be noted—in the 1950s—1960s (especially 1951, 1966) and in the 1980s (especially 1988). In the 1950–1960s, the main collections came from A.K. Skvortsov (850 estimated number of specimens/119 digitised), V.I. Sobolevsky (460/64), A.P. Khokhryakov (290/41) and in the 1980s from Konovalova (350/49), Smirnova (340/48) and Proskuryakova (340/47).

## Usage rights

### Use license

Other

### IP rights notes

This work is licensed under a Creative Commons Attribution (CC-BY) 4.0 Licence (http://creativecommons.org/licenses/by/4.0/). The licence covers images of the herbarium specimens deposited in https://plant.depo.msu.ru/ and available in GBIF, as well as their metadata.

## Data resources

### Data package title

MHA Herbarium: Eastern European collections of vascular plants

### Resource link

https://doi.org/10.15468/827lk2

### Alternative identifiers

https://www.gbif.org/dataset/af5f680a-e0cc-46c8-b623-ceeaab70aa9e, https://depo.msu.ru/ipt/resource?r=moscowmha

### Number of data sets

1

### Data set 1.

#### Data set name

MHA Herbarium: Eastern European collections of vascular plants

#### Data format

Darwin Core

#### Number of columns

1

#### Download URL

https://depo.msu.ru/ipt/resource?r=moscowmha

#### Description

**Data set 1.** 

Column label	Column description
MHA Herbarium: Eastern European collections of vascular plants	In 2017–2019, the Herbarium staff imaged the Moscow section (100%) and the Eastern European section (14.1%) of the MHA Herbarium. In total, 64,008 specimens were digitised (600 dpi images and key metadata). These data were published in the Moscow Digital Herbarium in 2019–2020 and fully available in GBIF. Based on these data, a detailed overview of the physical collections of these two sections is given in this data paper, as well as spatial, temporal and taxonomic description of the dataset. As of May 2020, 50,324 specimens from the MHA Herbarium have been georeferenced (78.6%) and 39,448 specimens have fully captured label transcriptions (61.6%).

## Additional information

**Collectors**

**1. Moscow section**. Full list of collectors consists of 823 surnames, including 127 people who collected more than 10 specimens. The list of top collectors of the Moscow section is given in Table 11, supplemented by the portrait galleries (Fig. 4 and Fig. 5).

The basis of the Moscow section was formed by ca. 2,000 specimens from D.P. Syreyshchikov and ca. 700 specimens from P.A. Smirnov, collected in 1920s and received from the Timiryazev Institute of Plant Physiology (Moscow).

The staff collected later accessions directly from the Garden (Ostankino in Moscow) and various areas across the Moscow Oblast – V.N. Voroshilov (1940–1950s), T.N. Evtyukhova (1940s), V.A. Shtamm (1940–1960s), G.P. Rysina (1960s) and B.M. Kulkov (1940–1950s). The donations of V.I. Sobolevsky (1950s), A.A. Nekrasov (1950–1960s), A.I. Manin (1960–1970s), A.P. Khokhryakov (1950–1960s) and others from different areas of the Moscow Region enriched the section as well.

In 1970–1990s, V.V. Makarov, M.S. Ignatov, A.N. Shvetsov, V.D. Bochkin, E.E. Gogina, and A.E. Matsenko made the largest collections across the Moscow Region due to the research missions of the Garden employees devoted to rare and endangered plant species, audit and organisation of the protected areas with a focus on the districts west of Moscow.

The Garden staff also intensively studied the alien flora of the Moscow Region. This resulted in the special collections by A.K. Skvortsov, V.V. Makarov, M.S. Ignatov and A.N. Shvetsov, expanded later by V.D. Bochkin assisted by S.R. Mayorov, Yu.A. Nasimovich and Yu.K. Vinogradova. Their collections became the basis of monographic reviews on the alien flora of the Moscow Region (Ignatov et al. 1990, Mayorov et al. 2012, Mayorov et al. 2020).

E.I. Kurchenko (Serpukhov District), N.M. Reshetnikova (Ruza District), V.B. Kuvaev (Znamenskoye near Moscow), Yu.A. Nasimovich with L.A. Deystfeldt (several districts) donated their collections from the Moscow Region (Skvortsov and Belyanina 2005). In the last decade, K.Yu. Teplov transferred large collections of rare plants from the locations across the Region.

**2. Eastern European section**. The collection consists mainly of specimens which the Garden staff collected during field trips since the 1950s. Initially, herbarium vouchers accompanied living plants and seeds collected in the wild for the displays of the Garden. This documentation activity was later supplemented by extensive floristic and taxonomic studies, conservation research and monitoring of alien species.

**2.1. Lower Volga Region.** The flora of the southeast of European Russia is the most fully represented regional flora of the Eastern European section. The Region known as Lower Volga includes Volgograd, Astrakhan and Saratov Oblasts and the Republic of Kalmykia. This is a predominantly semi-arid steppe region. The list of collectors includes 136 surnames (see top-collectors in Table 12 and Fig. 6), but for 53 people, only a single specimen have been digitised so far.

A.K. Skvortsov began his studies of the Lower Volga Region in the 1950s. In 1970–1990s, floristic expeditions were regular and the key collectors of that time were A.K. Skvortsov, A.E. Matsenko, V.V. Makarov, N.B. Belyanina, I.A. Shantser and V.D. Bochkin, as well as staff members of the Volgograd Pedagogical University (N.G. Volodina, V.A. Sagalaev and G.Yu. Klinkova). In 2010s, the collection activities were continued by N.Yu. Stepanova assisted by A.V. Kuvaev (Severtsov Institute) and I.N. Safronova (Komarov Institute) during their floristic studies of the Kuma-Manych depression and the Caspian Lowland.

A vast amount of material helped to critically assess the current state of the flora of the southeast of European Russia and with the publication of two volumes of the “Flora of the Lower Volga” (Skvortsov 2006, Reshetnikova 2018). The third volume of the series is expected in near future.

**2.2. Other areas.** The MHA Herbarium covers all regions of Eastern Europe within the former USSR with varying degree of completion. Table 13 shows the main collections from this territory, excluding Lower Volga. An additional gallery of top collectors is given in Fig. 7. Description and map of the curatorial areas used in the Moscow Digital Herbarium is available online (Seregin 2020b).

A large number of specimens from the **Central Forest-steppe Region** resulted from the recent floristic studies by staff member N.M. Reshetnikova (Belogorye Reserve, Belgorod Oblast) and graduate student A.K. Mamontov (Veidelevsky District, Belgorod Oblast). Collections by V.N. Voroshilov (Voronezh Oblast), V.V. Makarov (Tambov Oblast) and A.P. Khokhryakov (Penza Oblast) should also be acknowledged.

Collections from the **Western Region** (Smolensk and Bryansk Oblasts) were also made mainly by the herbarium staff (A.K. Skvortsov, V.V. Makarov and N.B. Belyanina). Yu.E. Alekseev from Moscow University donated his collections from Bryansk Oblast as a gift.

A large number of collections from the **Central Region** are associated with lengthy floristic studies by N.M. Reshetnikova in Kaluga Oblast expanded significantly by A.K. Krylov by the study of alien plants (Reshentnikova et al. 2010). In addition, the herbarium was enriched by the collections of V.D. Bochkin and V.V. Makarov from Kaluga, Tula and Vladimir Oblasts. Lesser collections were donated by V.I. Sobolevsky (Kaluga Oblast) and A.P. Seregin (Vladimir and Tula Oblasts).

The **West-Ukrainian Region** is represented mainly by collections from the Carpathians, donated by V.I. Sobolevsky and A.P. Khokhryakov and minor gatherings by the expedition headed by A.K. Skvortsov.

Major collections from the **Eastern Region** were donated by L.A. Utkin (Southern Urals), A.P. Khokhryakov and M.T. Mazurenko (Bashkiria). A.K. Skvortsov also collected a lot in the 1950s in the Denezhkin Kamen Reserve (Sverdlovsk Oblast) and Zlatoust (Chelyabinsk Oblast).

Important collections from other areas include:

- **Northern Region**—A.K. Skvortsov (Western Polar Urals, Khibiny), T.M. Smirnova (Karelia, Kola Peninsula), M.S. Ignatov (Arkhangelsk Oblast, Nenets Autonomous Okrug);
- **Rostov Oblast**—N.Yu. Stepanova (Kumo-Manch Depression and adjacent areas);
- **Western Siberia**—A.K. Skvortsov (Eastern Polar Urals);
- **Lithuania** and the **North-Ukrainian Region**—V.V. Makarov.

## Figures and Tables

**Figure 4a.**
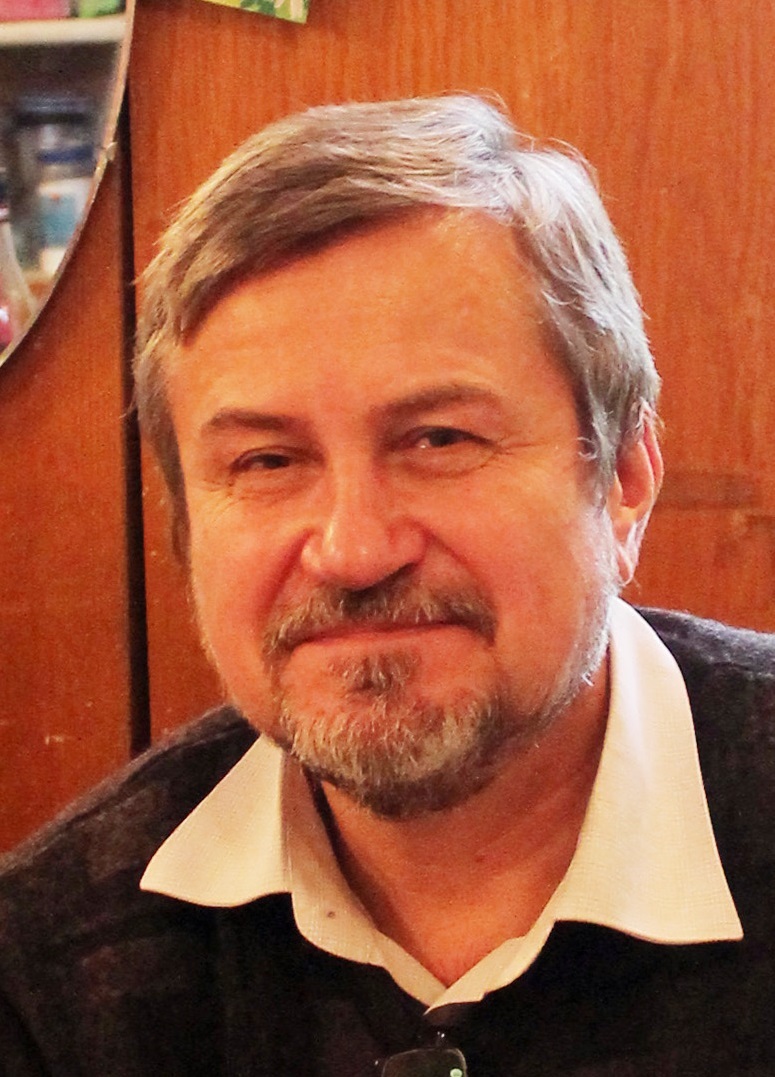
V.D. Bochkin

**Figure 4b.**
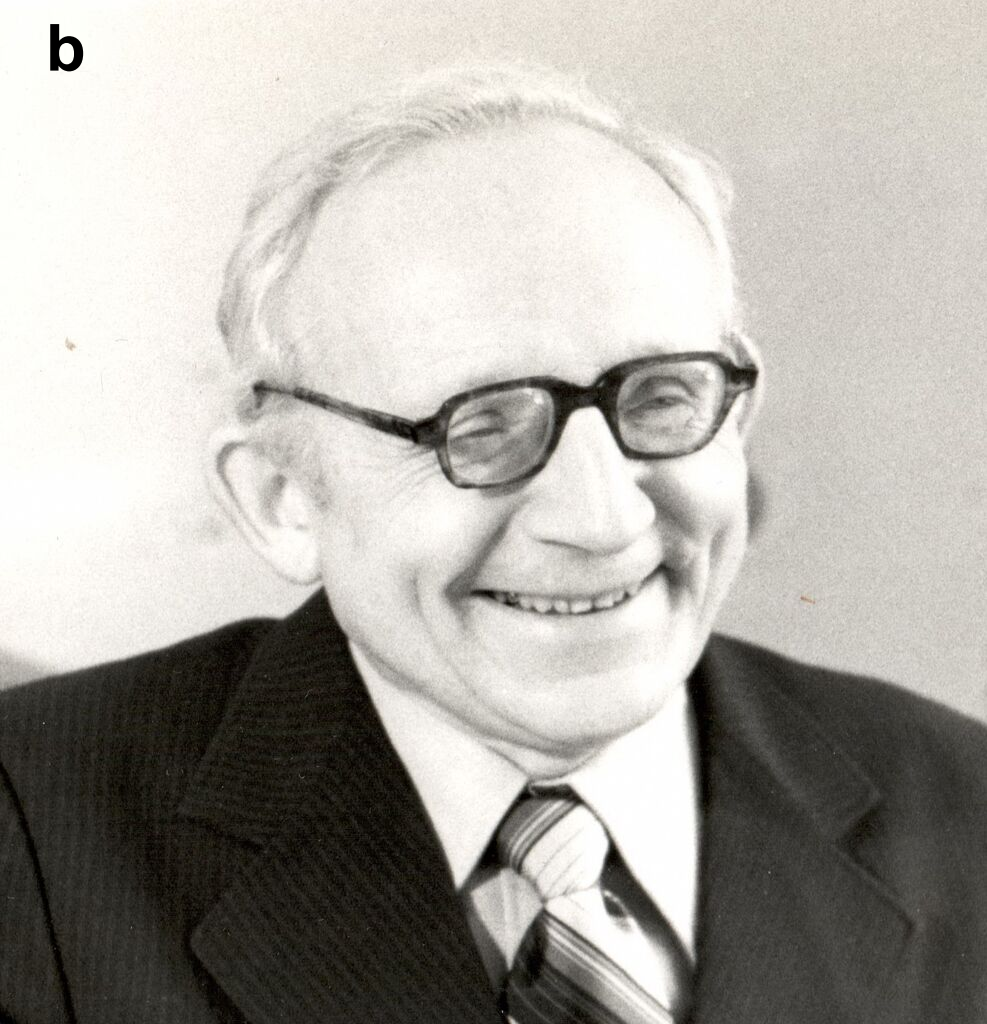
A.K. Skvortsov

**Figure 4c.**
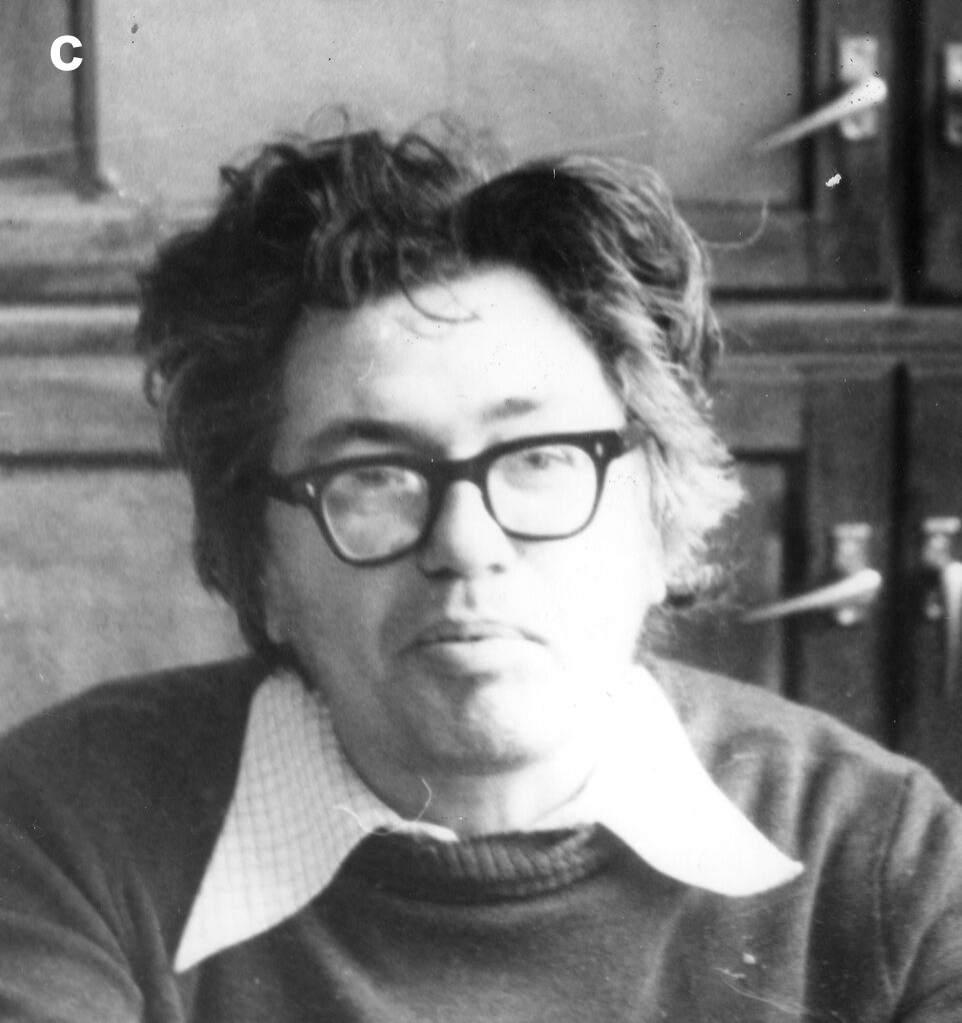
V.V. Makarov

**Figure 4d.**
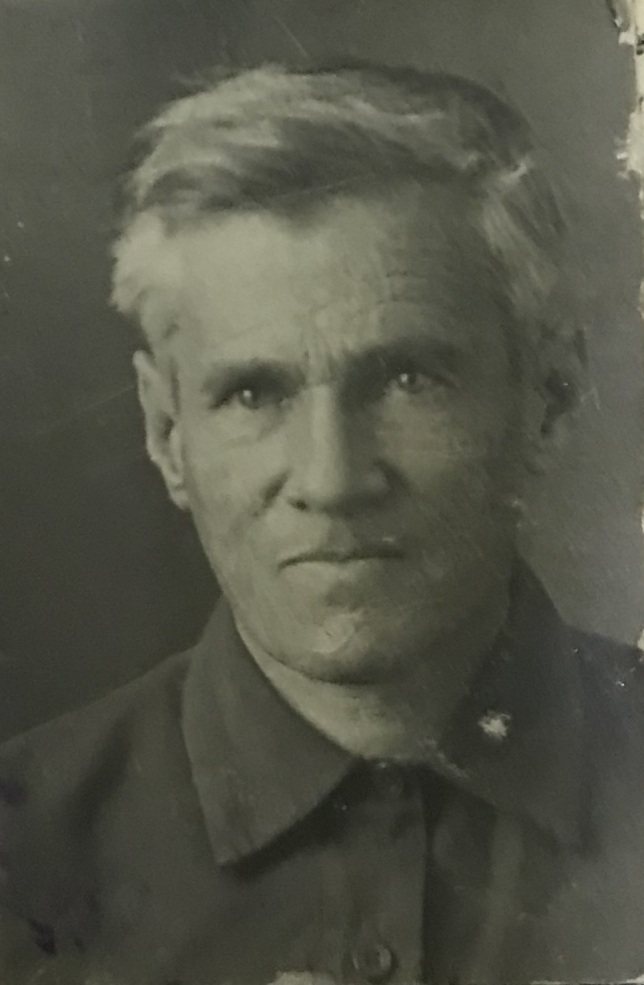
V.A. Shtamm

**Figure 4e.**
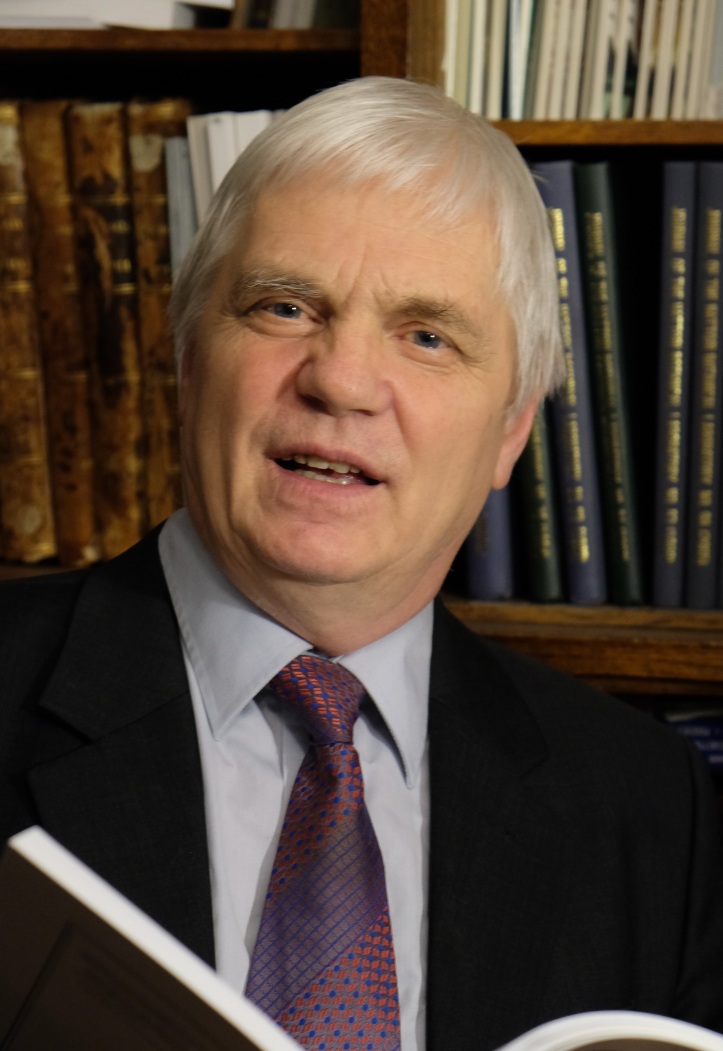
M.S. Ignatov

**Figure 4f.**
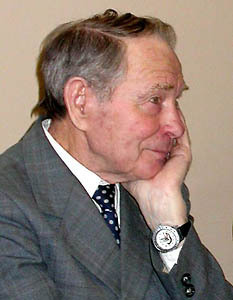
V.B. Kuvaev

**Figure 5a.**
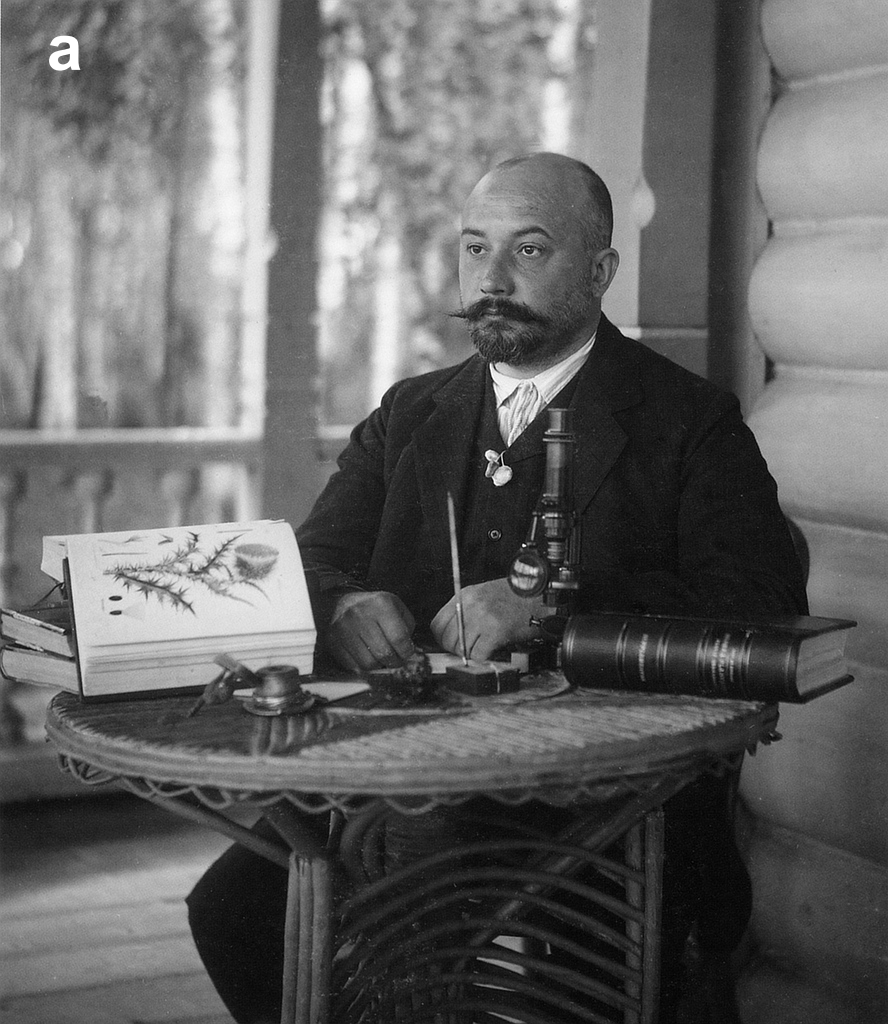
D.P. Syreyshchikov

**Figure 5b.**
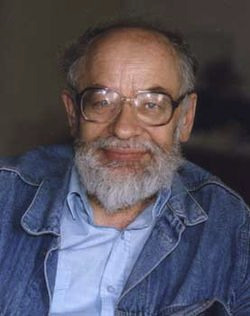
A.P. Khokhryakov

**Figure 5c.**
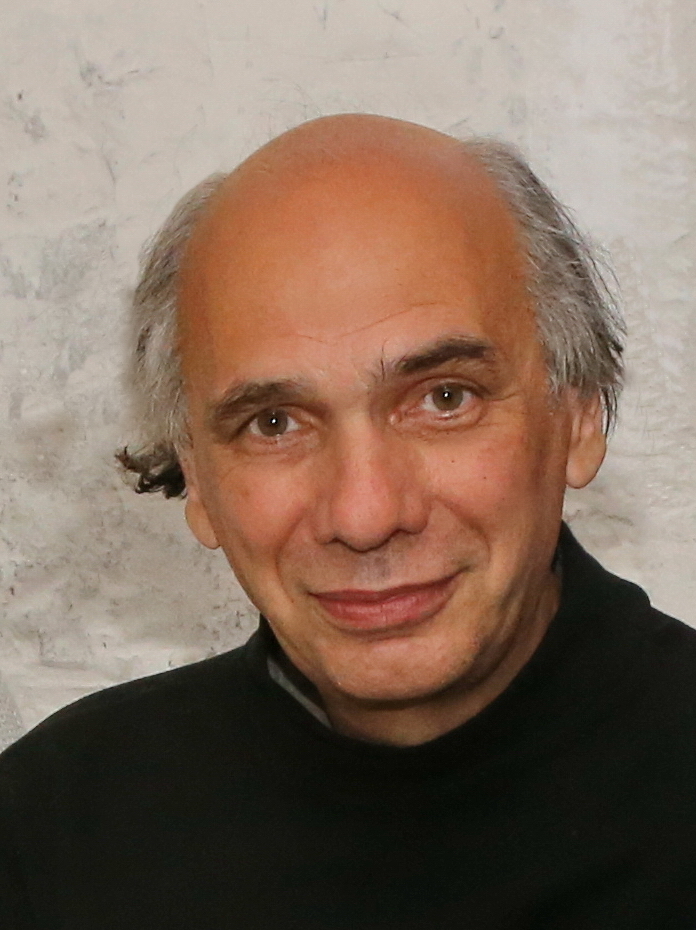
Yu.A. Nasimovich

**Figure 5d.**
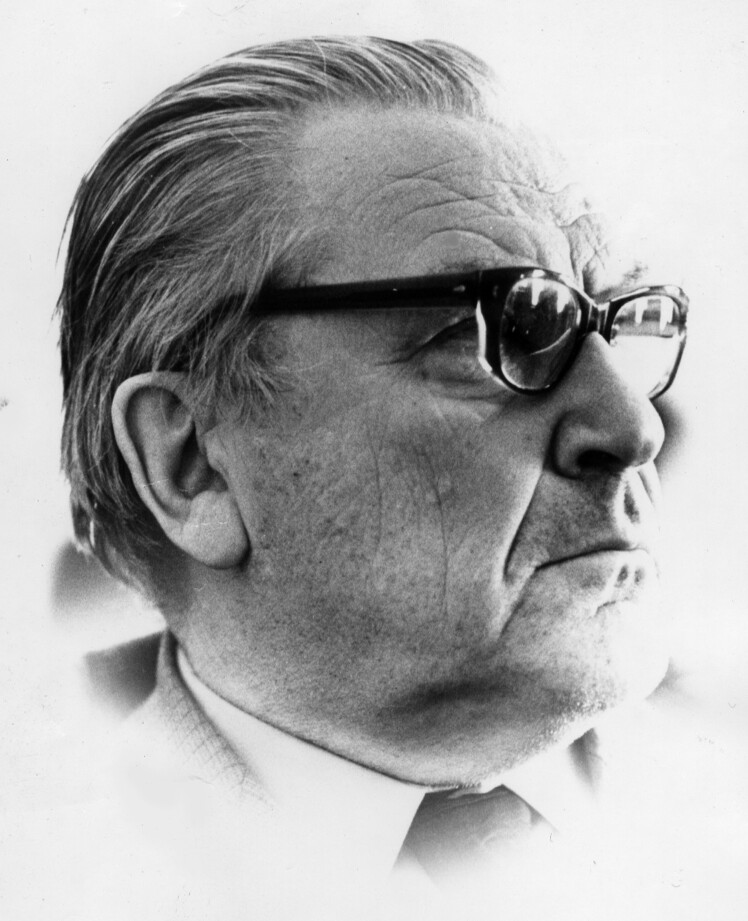
V.N. Voroshilov

**Figure 5e.**
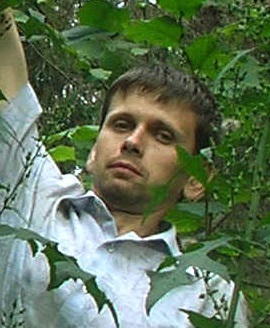
K.Yu. Teplov

**Figure 5f.**
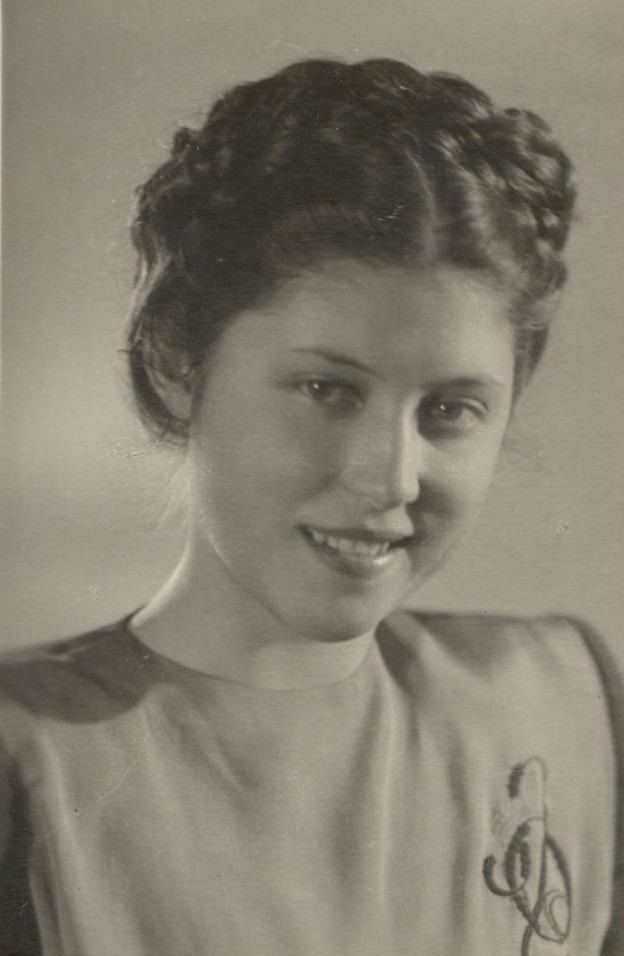
G.P. Rysina

**Figure 6a.**
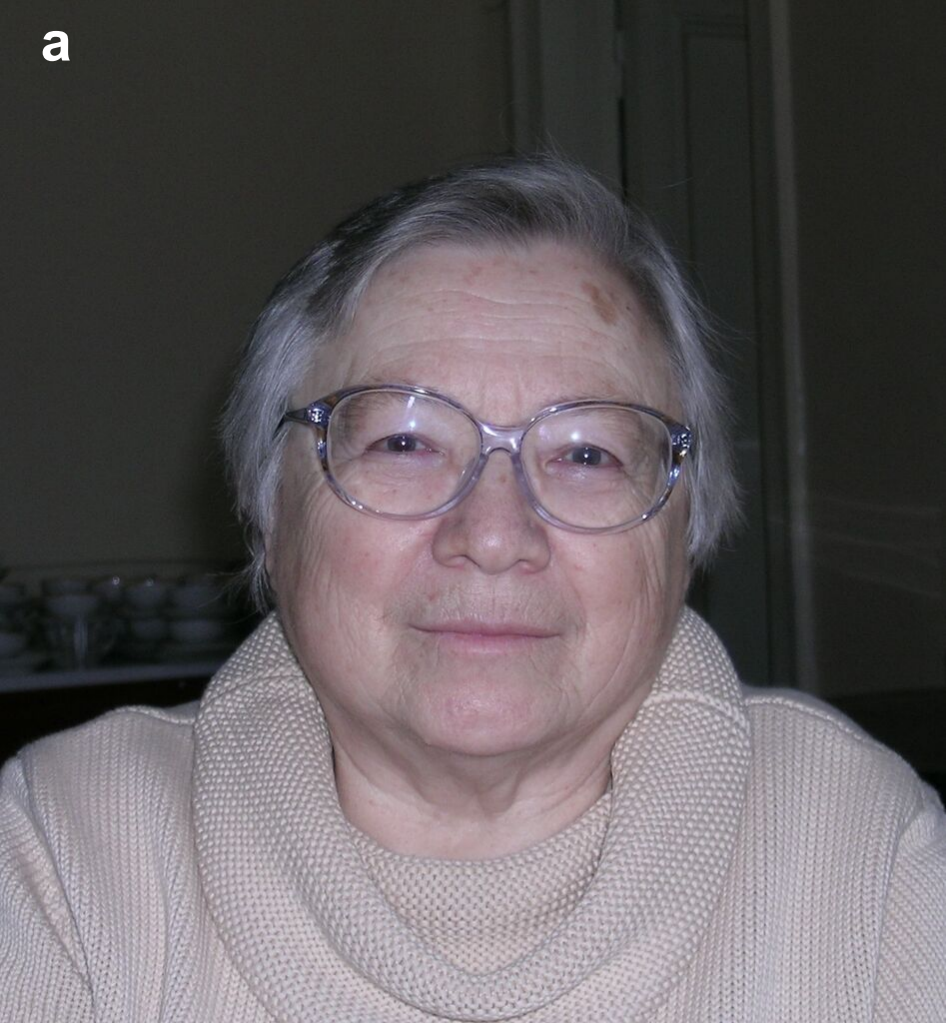
N.B. Belyanina

**Figure 6b.**
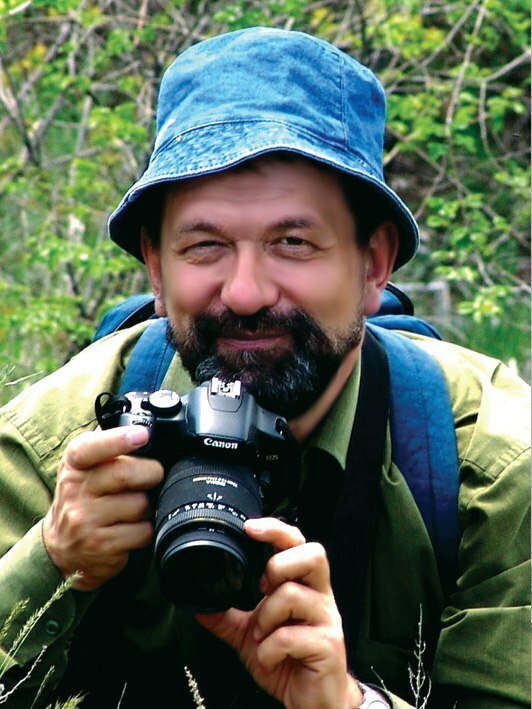
V.A. Sagalaev

**Figure 6c.**
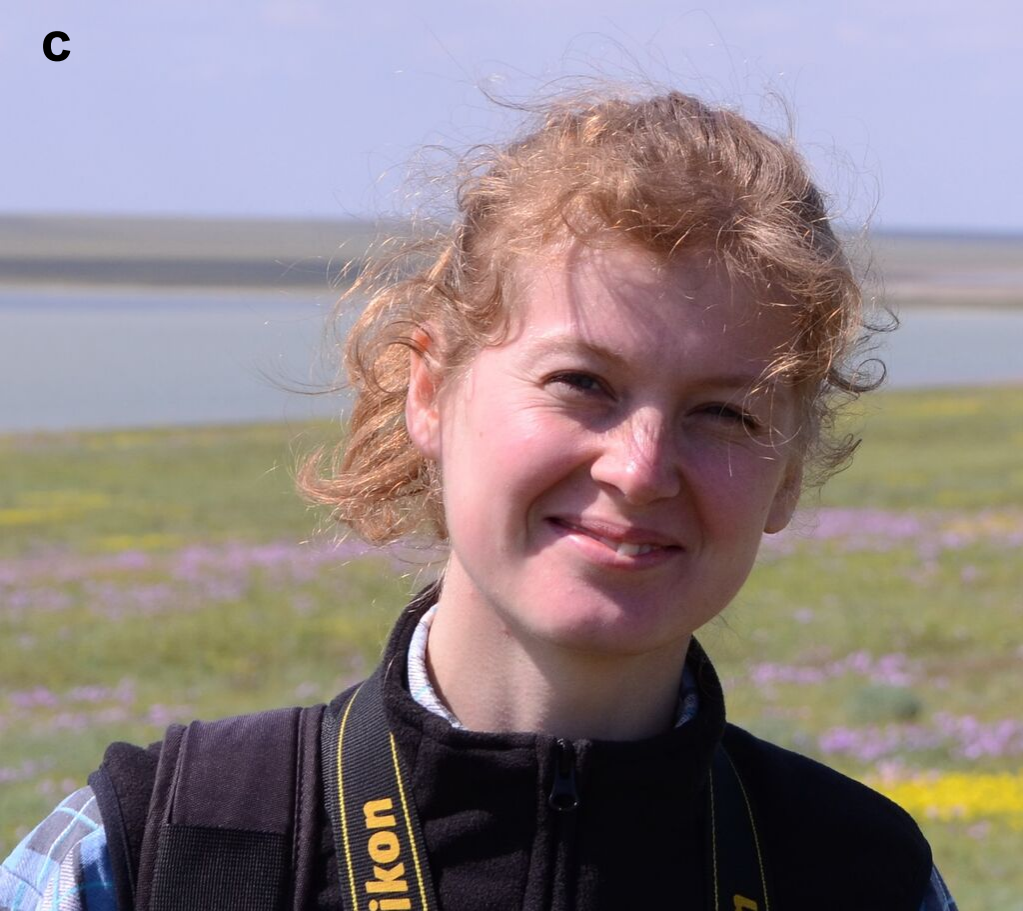
N.Yu. Stepanova

**Figure 6d.**
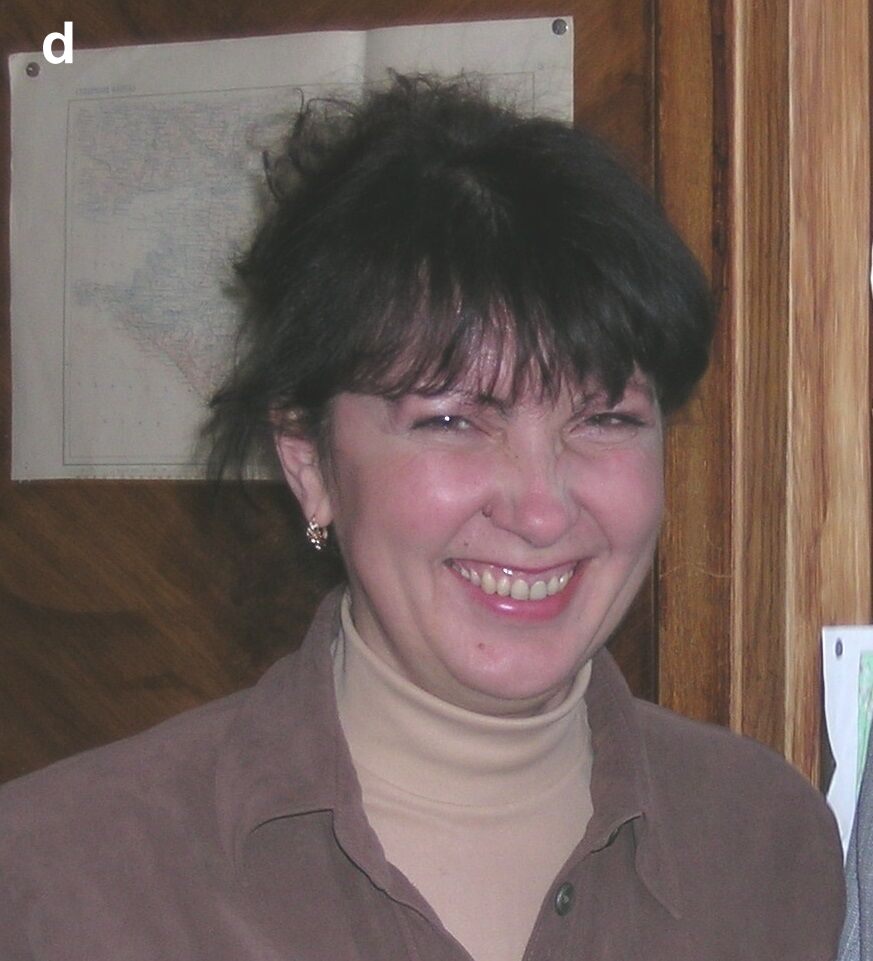
G.Yu. Klinkova

**Figure 6e.**
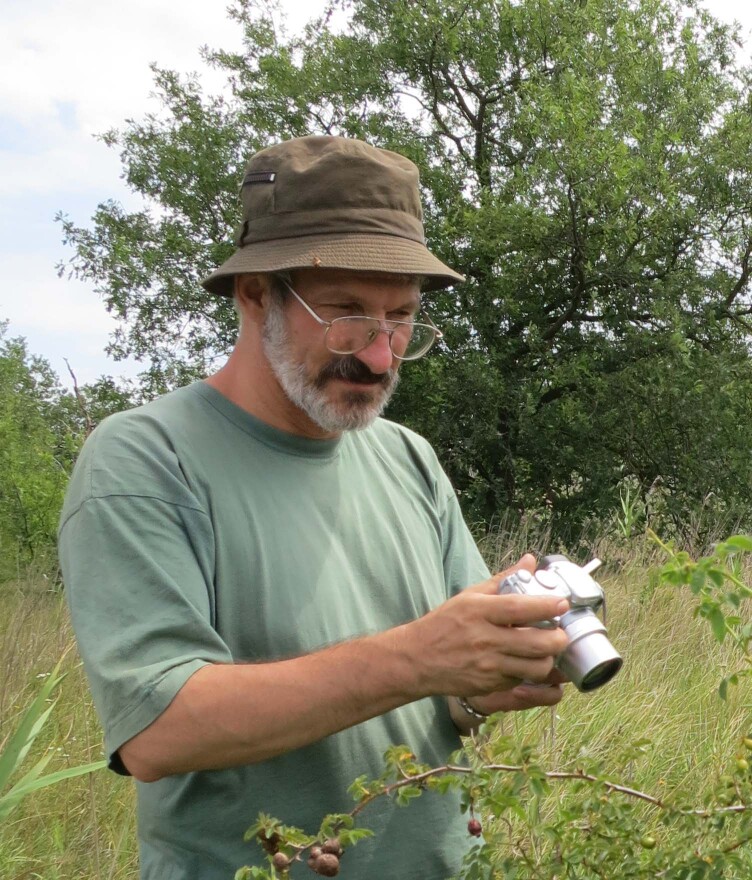
I.A. Shantser

**Figure 6f.**
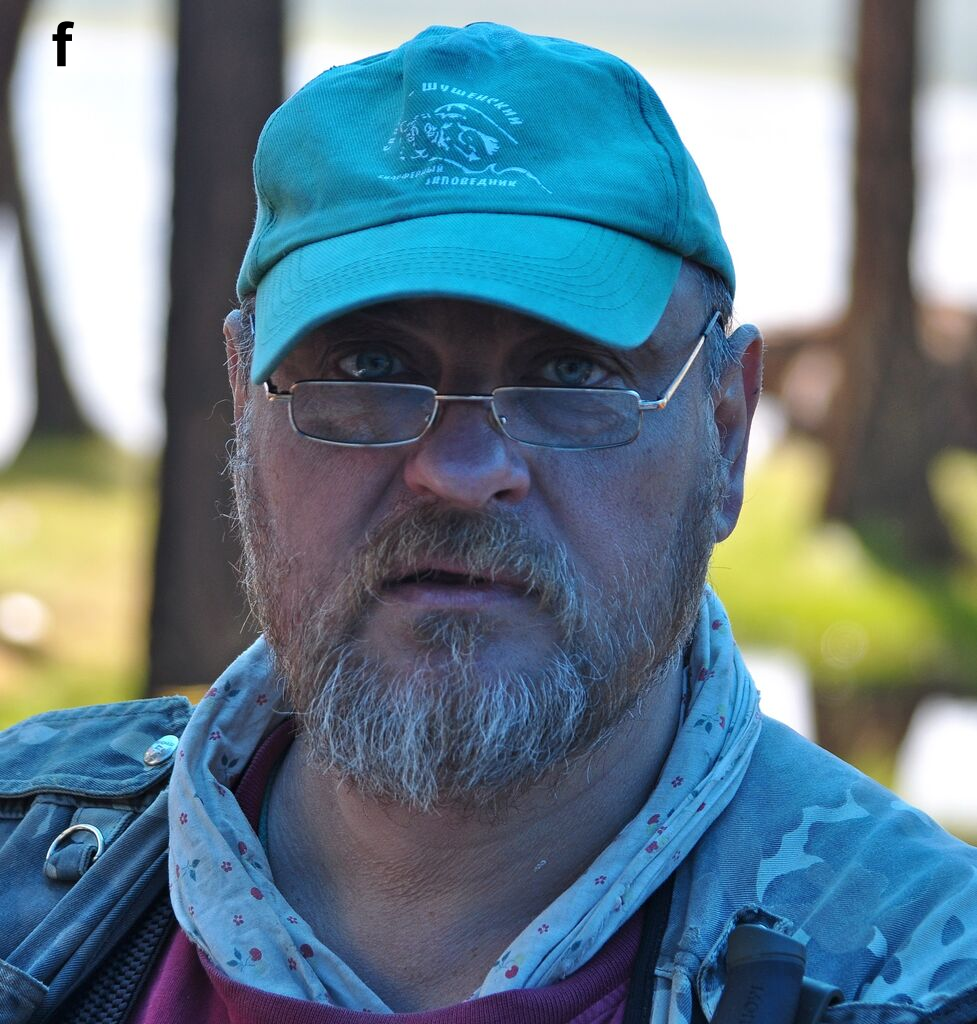
A.V. Kuvaev

**Figure 7a.**
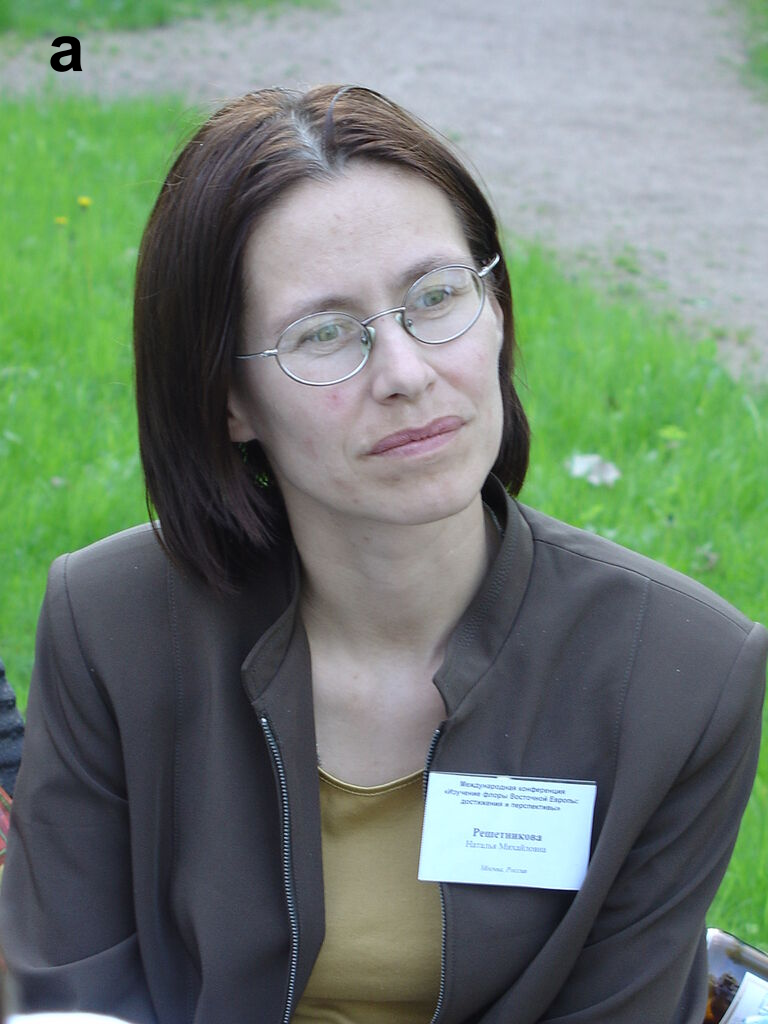
N.M. Reshetnikova

**Figure 7b.**
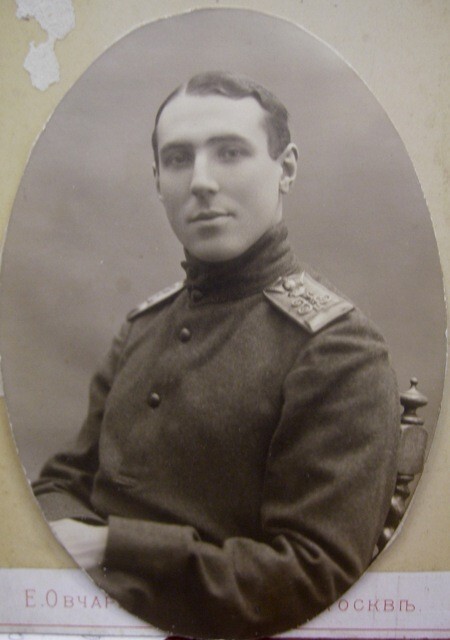
V.I. Sobolevsky

**Figure 7c.**
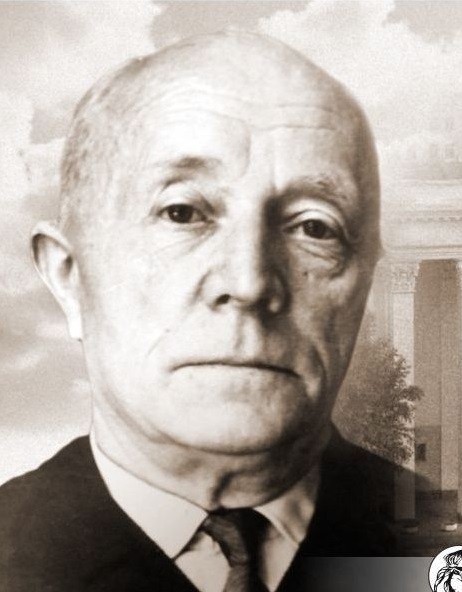
L.A. Utkin

**Figure 7d.**
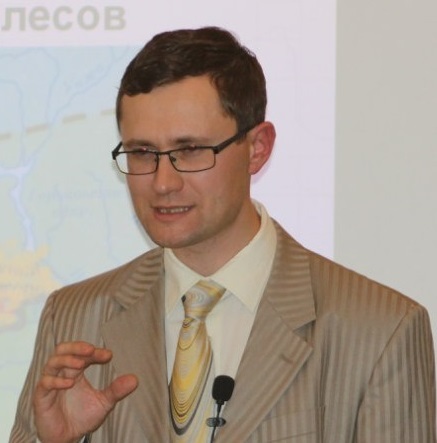
A.P. Seregin

**Figure 7e.**
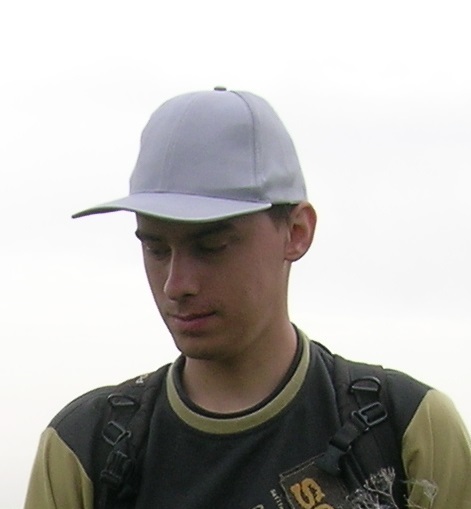
A.K. Mamontov

**Figure 7f.**
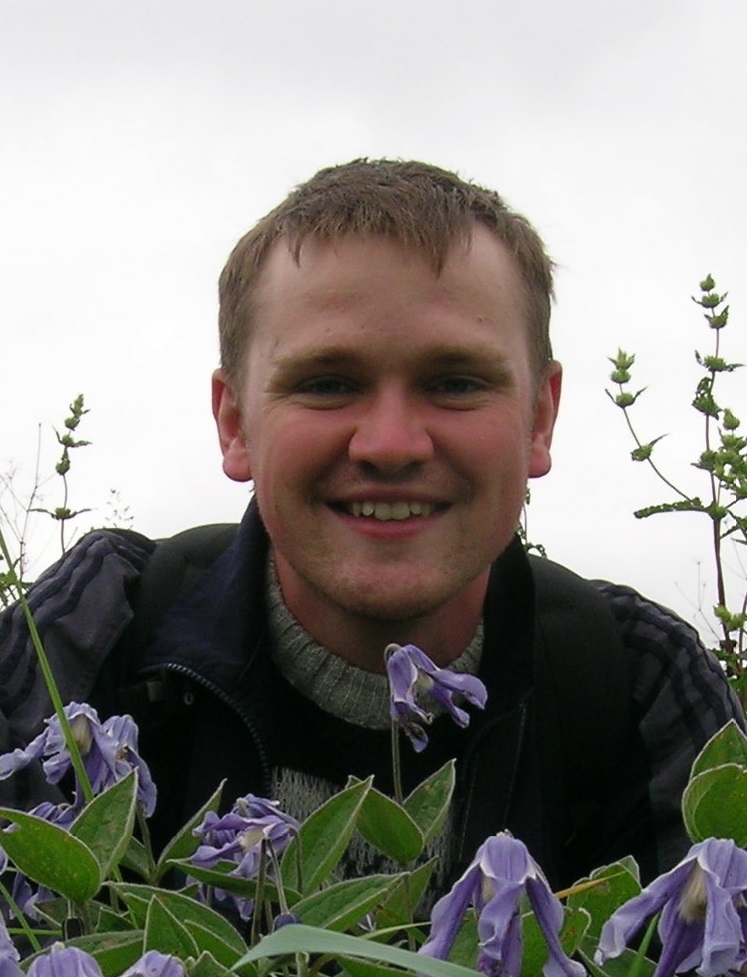
A.K. Krylov

**Table 1.** General overview of the MHA Herbarium collections.

	**Number of specimens**	**Proportion (%)**	**Mean annual growth (2014–2019)**
Eastern Europe	101,034	16.4	1,873
Moscow region	49,621	8.1	1,730
Crimea	17,915	2.9	24
Caucasus	52,553	8.5	202
Siberia and plains of Kazakhstan	60,903	9.9	691
Russian Far East	63,560	10.3	36
Middle Asia	52,557	8.5	389
General Herbarium	115,893	18.8	746
Herbarium of Introduction	23,981	3.9	213
Dendrological Herbarium	30,000	4.9	0
Type collection	1,424	0.3	0.5
Skvortsov’s personal herbarium	45,782	7.4	0
**All vascular plants**:	**615,223**	**100**	**5,906**
Bryophytes	70,000		ca. 2,000
Lichens (since 2019)	500		500
**Total**:	ca. 685,700		

**Table 2.** Collections of the MHA Herbarium from Eastern Europe by country.

**Rank**	**Country**	**Estimated total number of specimens**
1	Russia	131,420
2	Ukraine	12,410
3	Lithuania	1,640
4	Belarus	1,610
5	Kazakhstan	1,540
6	Estonia	1,290
7	Moldova	830
8	Latvia	390

**Table 3.** Collections of the MHA Herbarium from Eastern Europe by curatorial areas.

**Rank**	**Curatorial areas used in** **the Moscow Digital Herbarium**	**Number of digitised specimens**	**Estimated total** **number of specimens**
1	Moscow Region	49,744	49,744
2	Lower Volga Region	3,244	23,170
3	Central Region	1,812	12,940
4	Central Forest-steppe Region	1,459	10,420
5	Northern Region	1,259	8,990
6	Eastern Region	1,094	7,810
7	West-Ukrainian Region	954	6,810
8	Western Region	889	6,350
9	Northwest Region	690	4,930
10	North-Ukrainian Region	439	3,140
11	Middle Volga Region	435	3,110
12	South-Ukrainian Region	346	2,470
13	Rostov Oblast	231	1,650
14	Lithuania	229	1,640
15	Belarus	228	1,630
16	Western Kazakhstan	219	1,560
17	Estonia	180	1,290
18	Western Siberia	132	940
19	Moldova	116	830
20	Volga-Kama Region	87	620
21	Central forest Region	84	600
22	Latvia	54	390

**Table 4.** Top families of the Moscow section, MHA Herbarium.

**Rank**	**Family**	**Number of** **specimens**	**Rank**	**Family**	**Number of** **specimens**
1	Asteraceae	5,410	11	Apiaceae	1,334
2	Poaceae	5,075	12	Polygonaceae	1,279
3	Rosaceae	3,733	13	Plantaginaceae	1,109
4	Cyperaceae	3,195	14	Violaceae	1,064
5	Brassicaceae	2,518	15	Amaranthaceae	1,044
6	Fabaceae	2,007	16	Boraginaceae	1,023
7	Lamiaceae	1,954	17	Onagraceae	982
8	Ranunculaceae	1,689	18	Ericaceae	679
9	Caryophyllaceae	1,515	19	Orchidaceae	622
10	Salicaceae	1,437	20	Orobanchaceae	622

**Table 5.** Top genera of the Moscow section, MHA Herbarium.

**Rank**	**Genus**	**Number of specimens**	**Rank**	**Genus**	**Number of specimens**
1	* Carex *	2,561	11	* Pilosella *	494
2	* Salix *	1,108	12	* Potentilla *	470
3	* Viola *	1,064	13	* Juncus *	453
4	* Epilobium *	759	14	* Bromus *	448
5	* Ranunculus *	737	15	* Rumex *	439
6	* Veronica *	661	16	* Vicia *	438
7	* Alchemilla *	635	17	* Myosotis *	399
8	* Poa *	624	18	* Prunus *	396
9	* Galium *	589	19	* Stellaria *	387
10	* Campanula *	497	20	* Mentha *	383

**Table 6.** Top species of the Moscow section, MHA Herbarium.

**Rank**	**Species**	**Number of specimens**
1	*Carex nigra* (L.) Reichard	270
2	*Carex acuta* L.	224
3	*Mentha arvensis* L.	194
4	*Ranunculus cassubicus* L.	188
5	*Viola canina* L.	188
6	*Salix myrsinifolia* Salisb.	158
7	*Symphyotrichum salignum* (Willd.) G.L.Nesom	151
8	*Dryopteris carthusiana* (Vill.) H.P. Fuchs	147
9	*Calamagrostis canescens* (Weber) Roth	144
10	*Epilobium ciliatum* Raf.	144
11	*Chenopodium album* L.	141
12	*Valeriana officinalis* L.	133
13	*Carex rostrata* Stokes	131
14	*Epilobium roseum* Schreb.	131
15	*Glechoma hederacea* L.	130
16	*Persicaria lapathifolia* (L.) Gray	126
17	*Epilobium pseudorubescens* A.K. Skvortsov	125
18	*Polygonum aviculare* L.	125
19	*Alchemilla micans* Buser	122
20	*Rosa majalis* Herrm.	122

**Table 7.** Top families of the Eastern European section, MHA Herbarium (digitised specimens only).

**Rank**	**Families**	**Number of** **specimens**	**Rank**	**Families**	**Number of** **specimens**
1	Poaceae	8441	11	Cystopteridaceae	241
2	Potamogetonaceae	859	12	Hydrocharitaceae	160
3	Cyperaceae	761	13	Cupressaceae	156
4	Equisetaceae	460	14	Thelypteridaceae	140
5	Lycopodiaceae	443	15	Athyriaceae	130
6	Dryopteridaceae	428	16	Aspleniaceae	104
7	Typhaceae	339	17	Polypodiaceae	96
8	Rosaceae	313	18	Ephedraceae	88
9	Pinaceae	264	19	Juncaginaceae	75
10	Alismataceae	242	20	Ophioglossaceae	67

**Table 8.** Top genera of the Eastern European section, MHA Herbarium (digitised specimens only).

**Rank**	**Genera**	**Number of specimens**	**Rank**	**Genera**	**Number of specimens**
1	* Poa *	835	11	* Crataegus *	311
2	* Festuca *	806	12	* Elymus *	258
3	* Potamogeton *	678	13	* Puccinellia *	257
4	* Stipa *	624	14	* Glyceria *	236
5	* Bromus *	613	15	* Agropyron *	221
6	* Equisetum *	460	16	* Alopecurus *	215
7	* Dryopteris *	367	17	* Eriophorum *	208
8	* Agrostis *	335	18	* Lolium *	201
9	* Koeleria *	334	19	* Alisma *	187
10	* Calamagrostis *	327	20	* Anthoxanthum *	187

**Table 9.** Top species of the Eastern European section, MHA Herbarium (digitised specimens only).

**Rank**	**Species**	**Number of specimens**
1	*Festuca valesiaca* Schleich. ex Gaudin	193
2	*Festuca ovina* L.	157
3	*Koeleria pyramidata* (Lam.) P. Beauv.	153
4	*Stipa pennata* L.	145
5	*Festuca rubra* L.	140
6	*Dryopteris carthusiana* (Vill.) H.P. Fuchs	134
7	*Elymus repens* (L.) Gould	128
8	*Cystopteris fragilis* (L.) Bernh.	119
9	*Poa bulbosa* L.	119
10	*Bolboschoenus maritimus* (L.) Palla	116
11	*Crataegus monogyna* Jacq.	115
12	*Equisetum arvense* L.	111
13	*Juniperus communis* L.	111
14	*Spinulum annotinum* (L.) A. Haines	111
15	*Stipa lessingiana* Trin. et Rupr.	110
16	*Koeleria glauca* (Spreng.) DC.	106
17	*Poa palustris* L.	105
18	*Poa nemoralis* L.	100
19	*Stuckenia pectinata* (L.) Börner	98
20	*Bromus tectorum* L.	97

**Table 10.** Mean collection date of the MHA Herbarium holdings across the regions.

**Rank**	**Curatorial areas used** **in the Moscow Digital Herbarium**	**Mean collection year**	**Number of digitised** **specimens** **with collection date**
1	Rostov Oblast	2003	226
2	Lower Volga Region	1990	3,145
3	Central Region	1990	1,797
4	Central Forest-steppe Region	1984	1,444
5	Volga-Kama Region	1978	84
6	Western Region	1977	883
7	Western Siberia	1977	132
8	Moscow section	1976	49,560
9	Lithuania	1975	224
10	Western Kazakhstan	1975	211
11	Central Forest Region	1975	83
12	Northern Region	1971	1,242
13	Moldova	1971	116
14	Estonia	1969	178
15	South-Ukrainian Region	1968	334
16	Belarus	1968	221
17	West-Ukrainian Region	1967	923
18	North-Ukrainian Region	1964	432
19	Eastern Region	1959	1,073
20	Middle Volga Region	1959	424
21	Northwest Region	1953	678
22	Latvia	1932	54

**Table 11.** Top collectors of the Moscow section, MHA Herbarium.

**Rank**	**Collector**	**Number of specimens**
1	V.D. Bochkin (Fig. 4a)	9,868
2	A.K. Skvortsov (Fig. 4b)	4,170
3	V.V. Makarov (Fig. 4c)	3,888
4	V.A. Shtamm (Fig. 4d)	3,810
5	M.S. Ignatov (Fig. 4e)	2,745
6	V.B. Kuvaev (Fig. 4f)	2,659
7	D.P. Syreishchikov (Fig. 5a)	2,013
8	A.P. Khokhryakov (Fig. 5b)	1,674
9	Yu.A. Nasimovich (Fig. 5c)	1,463
10	V.N. Voroshilov (Fig. 5d)	1,368
11	K.Yu. Teplov (Fig. 5e)	1,185
12	G.P. Rysina (Fig. 5f)	949
13	E.E. Gogina	876
14	A.I. Manin	861
15	V.I. Sobolevsky	852
16	P.A. Smirnov	686
17	N.M. Reshetnikova	655
18	A.N. Shvetsov	579
19	A.A. Nekrasov	559
20	E.I. Kurchenko	554
21	T.N. Evtyukhova	494
22	B.M. Kulkov	468
23	L.A. Deistfeldt	311
24	A.E. Matsenko	291
25	N.K. Shvedchikova	284

**Table 12.** Top collectors of the Eastern European section, MHA Herbarium: Lower Volga Region.

**Rank**	**Collector**	**Number of digitised** **specimens (ca. 14%)**	**Estimated total** **number of specimens**
1	N.B. Belyanina (Fig. 6a)	542	3,870
2	V.A. Sagalaev (Fig. 6b)	413	2,950
3	N.Yu. Stepanova (Fig. 6c)	378	2,700
4	V.D. Bochkin (Fig. 4a)	376	2,690
5	G.Yu. Klinkova (Fig. 6d)	362	2,600
6	A.K. Skvortsov (Fig. 4b)	141	1,010
7	I.A. Shantser (Fig. 6e)	125	890
8	A.V. Kuvaev (Fig. 6f)	81	580
9	A.E. Matsenko	79	560
10	E.E. Gogina	73	520

**Table 13.** Top collectors of the Eastern European section, MHA Herbarium (excluding Lower Volga region).

**Rank**	**First collector**	**Curatorial areas** **used in the Moscow** **Digital Herbarium**	**Number of digitised specimens (ca. 14%)**	**Estimated total number of specimens**
1	N.M. Reshetnikova (Fig. 7a)	Central Forest-steppe Region	407	2,910
2	V.V. Makarov (Fig. 4c)	Western Region	381	2,720
3	N.M. Reshetnikova	Central Region	368	2,630
4	V.I. Sobolevsky (Fig. 7b)	West-Ukrainian Region	250	1,790
5	V.V. Makarov	Central Forest-steppe Region	219	1,560
6	A.P. Khokhryakov (Fig. 5b)	Eastern Region	206	1,470
7	L.A. Utkin (Fig. 7c)	Eastern Region	202	1,440
8	V.V. Makarov	Central Region	187	1,340
9	A.K. Skvortsov (Fig. 4b)	Northern Region	178	1,270
10	N.Yu. Stepanova (Fig. 6c)	Rostov Oblast	176	1,260
11	N.B. Belyanina (Fig. 6a)	Western Region	139	990
12	A.P. Seregin (Fig. 7d)	Central Region	132	940
13	V.D. Bochkin (Fig. 4a)	Central Region	127	910
14	A.K. Skvortsov	Western Siberia	115	820
15	V.N. Voroshilov (Fig. 5d)	Central Forest-steppe Region	111	790
16	A.K. Mamontov (Fig. 7e)	Central Forest-steppe Region	111	790
17	A.P. Khokhryakov	West-Ukrainian Region	110	790
18	A.K. Skvortsov	Eastern Region	107	760
19	A.K. Skvortsov	Western Region	106	760
20	V.V. Makarov	Lithuania	106	760
21	A.V. Krylov (Fig. 7f)	Central Region	106	760
22	Yu.E. Alekseev	Western Region	99	710
23	V.V. Makarov	North-Ukrainian Region	97	690
24	A.K. Skvortsov	West-Ukrainian Region	93	660
25	A.P. Khokhryakov	Central Forest-steppe Region	84	600
26	V.I. Sobolevsky	Central Region	84	600
27	T.M. Smirnova	Northern Region	80	570
28	M.S. Ignatov	Northern Region	78	560
